# Microscopic Polyangiitis With Selective Involvement of Central and Peripheral Nervous System: A Case Report

**DOI:** 10.3389/fneur.2020.00269

**Published:** 2020-04-28

**Authors:** Federica Arienti, Giulia Franco, Edoardo Monfrini, Alessandro Santaniello, Nereo Bresolin, Maria Cristina Saetti, Alessio Di Fonzo

**Affiliations:** ^1^Neuroscience Section, Department of Pathophysiology and Transplantation, Fondazione IRCCS Ca' Granda Ospedale Maggiore Policlinico, Milan, Italy; ^2^Referral Center for Systemic Autoimmune Diseases, Fondazione IRCCS Ca' Granda Ospedale Maggiore Policlinico, Milan, Italy

**Keywords:** microscopic polyangiitis, vasculitis, central pain, peripheral neuropathy, allodynia, foot drop, anti-neutrophil cytoplasmic antibodies specific for myeloperoxidase (MPO-ANCA)

## Abstract

**Background:** Microscopic polyangiitis (MPA) is a necrotizing vasculitis that affects predominantly small-sized vessels in many organ systems. The disease generally causes glomerulonephritis, pulmonary damage, arthritis, and neuropathy. An exclusive involvement of both central nervous system (CNS) and peripheral nervous system (PNS) is extremely rare.

**Case Presentation:** A 62-year-old woman was admitted to our hospital with a 3 months history of right foot drop, recently complicated by intense myalgia, arthralgia, and allodynia to tactile, vibratory, and pressure stimuli. Since blood tests revealed elevated inflammatory indexes, we suspected either infectious or immune-mediated disorders. Chest radiograph, blood culture series, and echocardiogram revealed normal findings, while urinalysis showed a bacterial infection that was successfully treated. The neurophysiological findings were compatible with multiple mononeuritis, and a brain MRI evidenced ischemic lesions of both basal ganglia and thalamus. A wide-spectrum autoantibody assay revealed the presence of high-titer perinuclear anti-neutrophil cytoplasmic antibodies specific for myeloperoxidase (MPO-ANCA). According to these findings, the diagnosis of MPA was made, and the patient was successfully treated with intravenous (IV) methylprednisolone, followed by two doses of rituximab.

**Conclusions:** An assessment of both CNS and PNS should be included in the diagnostic evaluation of MPA. The involvement of the PNS may raise the risk of a relapsing course and treatment failure, therefore it should be considered in the choice of induction and maintenance therapy.

## Background

Microscopic polyangiitis (MPA) is a necrotizing vasculitis that affects small- and some medium-sized vessels, often associated with perinuclear anti-neutrophil cytoplasmic antibodies specific for myeloperoxidase (MPO-ANCA) (1).

In most cases, MPA presents as rapidly progressive glomerulonephritis, pulmonary capillaritis, and general symptoms, including fatigue, fever, anorexia, muscle, and joint pain. The involvement of the peripheral nervous system (PNS) is also possible and not infrequent: distal symmetrical polyneuropathy and mononeuritis multiplex occur in more than a third of cases (2). Instead, central nervous system (CNS) involvement is less frequently described and generally consists of intracerebral or subarachnoid hemorrhage (3), pachymeningitis (4), and ischemic strokes (5).

Here we report a case of MPA patient who presented with an exclusive involvement of the nervous system, both peripherical and central. The patient provided her written informed consent for the publication of this case report and any accompanying images.

## Case Presentation

A 62-year-old woman was admitted to our hospital with a 3-month history of right foot drop, generalized weakness, apathy, and weight loss (4 kg), recently complicated by the development of diffuse pain with intense myalgia and arthralgia, unresponsive to analgesics.

Patient's history revealed a previous diagnosis of fibromyalgia and positivity for rheumatoid factor without anti-cyclic citrullinated peptide antibodies. Despite periodic chronic widespread pains, the patient has always been in good health.

Preliminary blood tests showed high values of C-reactive protein (CRP; 20.24 mg/dl; normal <0.5 mg/dl) and elevated white blood cell count (WBC; 17.350/mm^3^).

A neurologic examination confirmed the distal motor deficit of the right leg with muscle strength evaluated as 1/5 in right foot dorsiflexors and as 2/5 in plantar flexors on the Medical Research Council (MRC) scale. Hypoesthesia to pinprick and touch in a sock and glove distribution was detected. Deep tendon reflexes were present and symmetrical, except for right ankle reflex, which could not be elicited. Gait was slightly unsteady and typically stepping because of the foot drop. The patient showed a severe anxious state and an abnormal painful reaction to sensory stimuli, configuring a condition of hyperpathia and allodynia. Therefore, an analgesic therapy with transdermal fentanyl was started.

The subacute/acute onset of symptoms and the high level of inflammatory markers would suggest either infectious or immune-mediated disorders. Chest radiograph excluded parenchymal infiltrates or pleural effusions. Blood culture series were negative. Echocardiogram was within normal limits and showed no vegetations. The urinalysis demonstrated hematuria with high levels of bacteria and leukocyte esterase; a subsequent urine culture confirmed an *Escherichia coli* infection that was successfully treated according to the antibiogram. Despite this therapy, the inflammatory markers remained significantly elevated [CRP 20.12 mg/dl; WBC 15.300/mm^2^; erythrocyte sedimentation rate (ESR) 89 mm] and a minimal hematuria persisted in the 2 days following the end of antibiotic therapy.

Of note, a progressive bilateral wrist drop appeared 3 days after hospital admission.

Electromyography (EMG) revealed the absence of the sensory nerve action potentials (SNAPs) in the left median nerve and a reduction of conduction speed in the right median nerve. The amplitude of the compound muscle action potential (CMAP) was reduced in the left median nerve and, more markedly, in the right peroneal nerve, with preserved conduction velocity. Both CMAP amplitude and conduction velocity were reduced in the right ulnar nerve distal to the elbow. Needle EMG revealed active denervation in the right tibialis anterior muscles, right medial twin, short head of the right biceps femoris muscle, right abductor digiti minimi, and left common extensor of the fingers. Those findings were compatible with a marked sensory–motor axonal suffering in active phase, with a multi-neuropathic distribution.

**Figure 1:**
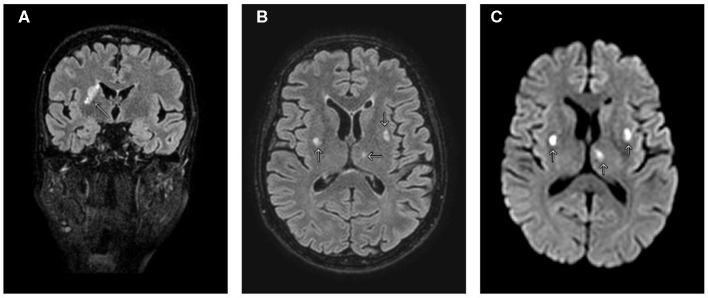
MRI of the brain. **(A)** T2/fluid-attenuated inversion recovery (FLAIR) coronal view shows hyperintensity in the right caudate, internal capsule, and putamen. **(B)** T2/FLAIR axial view shows additional lesions involving left thalamus and lenticular nuclei. **(C)** Diffusion-weighted imaging axial view shows three lesions with restricted diffusion. Arrows indicate CNS lesions.

Despite opioid analgesic therapy, the patient continued to suffer from widespread pain, with marked allodynia to tactile, vibratory, and pressure stimuli. Assuming a central origin of the pain, brain MRI was performed, revealing hyperintense lesions in the left thalamus, right caudate, and lenticular nuclei on T2/fluid-attenuated inversion recovery (FLAIR) images (Figures 1A,B), characterized by restricted diffusion (Figure 1C), without edema nor contrast enhancement.

The cerebrospinal fluid (CSF) composition was normal; real-time PCR assay for pathogens associated with CNS infection [herpes simplex virus (HSV)1, HSV2, varicella zoster virus (VZV), human herpesvirus (HHV)6, cytomegalovirus (CMV), enterovirus, parechovirus, *Neisseria meningitidis, Haemophilus influenzae, Streptococcus pneumoniae, E. coli, Listeria monocytogenes*, and *Cryptococcus neoformans*] was negative. Oligoclonal bands were absent in serum and CSF.

Serology revealed the presence of high-titer MPO-ANCA (278.30 UI/ml; reference <5.97 UI/ml). All the other autoantibodies tested [c-ANCA, anti-dsDNA, lupus anticoagulant, anti-phospholipid, autoantibodies to thyroid peroxidase (anti-TPO), autoantibodies to thyroglobulin (anti-Tg), autoantibodies to mitochondrial antigen (AMA), liver kidney microsome (LKM), anti-smooth muscle antibodies (ASMA), extractable nuclear antigen (ENA), anti-cyclic citrullinated peptide antibody (anti-CCP), anti-Scl70, anti-pmScl, anti-PL7, anti-Jo1, anti-signal recognition particle (anti-SRP)-S4, anti-Mi2, anti-Ku, anti-PL12] resulted negative.

MPO-ANCAs are autoantibodies associated to different types of small vessel vasculitis, like MPA, and eosinophilic granulomatosis with polyangiitis.

Considering the clinical presentation and the absence of eosinophilia, the patient was diagnosed with MPA.

MPA usually involves small vessels, but in order to exclude the involvement of medium vessels, we performed a magnetic resonance angiography of extracranial carotid arteries and intracranial vessels, which resulted negative.

In order to investigate the possibility of multi-organ involvement, additional testing was conducted. Pulmonary CT scan was unremarkable; urinary sediment examination, creatinine, and BUN were normal.

The patient was started on steroidal therapy with intravenous (IV) methylprednisolone 1 g for 5 days, followed by oral tapering with prednisone. After this treatment, peripheral sensitivity improved, inflammatory markers lowered (CRP 2.99 mg/dl; WBC 11.620/mm^2^), and anti-MPO antibodies started to decrease (209.2 UI/ml). However, foot and wrist drop minimally improved. Therefore, we chose to complete the induction therapy with a single dose of rituximab (1 g), followed by a second dose after 2 weeks, which led to a significant clinical improvement of hands motor deficit and also to a further decrease in the antibodies level (49.4 UI/ml).

After discharge from the hospital, the patient started neurorehabilitation and has since undergone neurophysiological, neuroradiological, and immuno-rheumatologic follow-up.

In the following 6 months, anti-MPO antibodies continued to decrease, the foot drop progressively resolved, and the patient returned to be completely asymptomatic.

**Figure 2:**
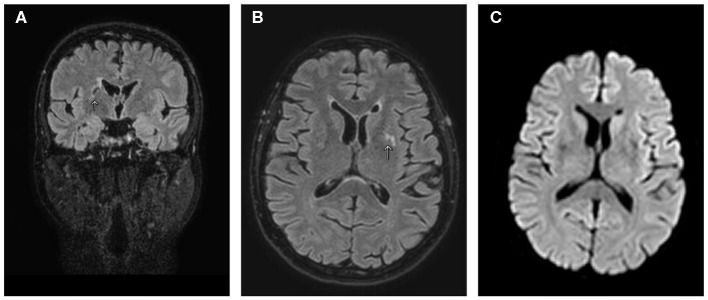
MRI of the brain after 3 months of follow-up. T2/fluid-attenuated inversion recovery (FLAIR) coronal **(A)** and axial **(B)** views show size reduction of the pre-existing lesions in the basal ganglia, thalamus, and internal capsule. **(C)** Diffusion-weighted imaging axial view showing no signal alteration. Arrows indicate CNS lesions.

A brain MRI performed 3 months after admission showed size reduction of the pre-existing lesions without further alterations in signal intensity nor restriction in the diffusion-weighted sequences (Figure 2).

## Discussion and Conclusions

Our patient presented an almost exclusive involvement of the nervous system: she developed a typical multiple mononeuritis, while the ischemic lesions of both basal ganglia and thalamus witnessed the lenticulostriate arteries involvement. The severe allodynia may have multiple causes: CNS lesions may account for a thalamic pain syndrome or central post-stroke pain (6), as well as vasculitis-associated neuropathy, and myalgia may also contribute to the hyperpathic syndrome.

Having excluded other diseases, the diagnosis of MPA was achieved because of MPO positivity and compatible clinical features using the European Medicines Agency (EMA) algorithm (7). Despite the major and almost isolated involvement of the nervous system, a minimal renal inflammation cannot be completely ruled out since the patient suffered from a slight hematuria.

The 2012 Revised International Chapel Hill criteria (8) could not be completely fulfilled, standing the absence of lung involvement, evident renal vasculitis, and histology proving necrotizing vasculitis.

Indeed, the classification of ANCA-associated small-vessel vasculitis remains complex in actual clinical practice. As a matter of fact, nearly 50% of patients are classified according to ANCA specificities without being able to fulfill the current diagnostic criteria (9).

We decided not to perform a nerve biopsy because the ANCA+ serology and the treatment response strongly suggested the diagnosis; besides, considering the segmental nature of the vasculitis, serial nerve sections should have been performed to obtain a good sensitivity (10). However, in the absence of such a significant antibody positivity, a nerve biopsy would have been recommended.

An isolated involvement of both CNS and PNS is exceptional in MPA; in a Spanish series with 167 MPA patients, neurological involvement was detected in 30.5% of patients, of whom only 5.4% had cerebrovascular accidents (11). Ito et al. (12) reported a case of ANCA-associated vasculitis causing bilateral cerebral infarction without renal and respiratory dysfunction, but in absence of PNS involvement. To our knowledge, only Sassi et al. (13) described a 48-year-old man who developed mononeuritis multiplex and massive cerebral hematoma apparently without any other systemic damage. Other cases reported in literature describe patients with hemorrhagic involvement of CNS accompanied by glomerulonephritis (14–16).

Treatment for MPA consists of IV prednisone in the acute setting, while cytotoxic drugs (e.g., cyclophosphamide, azathioprine), are chosen for maintenance therapy (17). Rituximab is another Food and Drug Administration (FDA)-approved option in combination with glucocorticoids and has been shown to be more effective than azathioprine for maintenance of remission (18). According to literature evidences, MPA patients with mononeuritis multiplex require more intensive treatments, since the risk of relapsing course and treatment failure is higher (19).

In conclusion, the diagnostic evaluation of MPA should include an assessment of both CNS and PNS, considering their relatively high frequency of involvement. Patient's characteristics, disease severity, and high risk of relapsing course should be considered in the choice of induction and maintenance therapy.

## Author's Information

FA and EM are resident physicians in neurology. GF, NB, MS, and AD are senior physicians in neurology. AS is a senior physician in immuno-rheumatology.

## Ethics Statement

The patient provided her written informed consent for the publication of this case report and any accompanying images.

## Author Contributions

FA took care of the patient, evaluated data, and was a major contributor in writing the manuscript. GF and EM took care of the patient, evaluated data, and reviewed the manuscript. AS analyzed and interpreted data regarding the immunological aspects of the disease. NB and MS took care of the patient and evaluated data. AD evaluated data, suggested the case report, and reviewed the manuscript. All authors read and approved the final manuscript.

## Conflict of Interest

The authors declare that the research was conducted in the absence of any commercial or financial relationships that could be construed as a potential conflict of interest.
